# Off-label use of IV t-PA in patients with intracranial neoplasm and cavernoma

**DOI:** 10.1177/1756285617753423

**Published:** 2018-02-05

**Authors:** Christopher Jan Schwarzbach, Anne Ebert, Michael G. Hennerici, Eva Neumaier-Probst, Michael Platten, Marc Fatar

**Affiliations:** Department of Neurology, UniversitätsMedizin Mannheim, University of Heidelberg, Theodor-Kutzer-Ufer 1-3, 68167 Mannheim, Germany; Department of Neurology, UniversitätsMedizin Mannheim, University of Heidelberg, Mannheim, Germany; Department of Neurology, UniversitätsMedizin Mannheim, University of Heidelberg, Mannheim, Germany; Department of Neuroradiology, UniversitätsMedizin Mannheim, University of Heidelberg, Mannheim, Germany; Department of Neurology, UniversitätsMedizin Mannheim, University of Heidelberg, Mannheim, Germany; Department of Neurology, UniversitätsMedizin Mannheim, University of Heidelberg, Mannheim, Germany

**Keywords:** brain neoplasms, cavernoma, stroke, thrombolytic therapy, treatment outcome

## Abstract

**Background:**

The safety of systemic thrombolysis in patients with intracranial tumor and cavernoma are unknown. So far evidence is limited to a number of case reports and few case series or unspecified data based on population-based analysis. Our aim was to comprehend the risk of systemic thrombolysis in these patients.

**Methods:**

Patients with additional evidence of intracranial tumor or cavernoma who received IV tissue plasminogen activator (t-PA) treatment at our comprehensive stroke center over a period of 7 years were identified in our stroke database and compared to the same number of matched control subjects without any evidence of intracranial tumor and cavernoma. Clinical history and imaging patterns before and after t-PA therapy were individually reviewed for each patient.

**Results:**

Thirty-four patients with additional evidence of meningioma (19/34), cavernoma (13/34) or malignant intracranial neoplasm (2/34) were identified. The incidence of secondary intracranial hemorrhage observed showed no difference between control subjects (9/34, 26%) and patients (6/34, 18%; *p* = 0.56). Symptomatic hemorrhage in patients with meningioma or cavernoma could not be observed. Likewise, the prevalence of stroke mimics showed no difference between patients (8/34, 24%) and control subjects (5/34, 15%; *p* = 0.54). However, both patients with malignant intracranial neoplasm presented with a stroke mimic and intracranial hemorrhage was observed in one of them.

**Conclusions:**

In compliance with existing evidence, treatment in patients with meningioma and cavernoma appears to be safe and reasonable, while the therapy should be avoided in patients with malignant intracranial neoplasm with blood–brain barrier disruption.

## Background

Intracranial tumors, especially of benign character like meningioma, and cavernoma are a relatively frequent secondary finding and, like systemic cancer, may occur in coincidence with acute ischemic stroke.^1,2^ Nevertheless, the safety of systemic thrombolysis in patients with intracranial tumor and cavernoma are unknown. So far evidence is limited to a number of case reports and few case series.^3–13^ Based upon these reports, systemic thrombolysis has been repeatedly discussed as a feasible option for selected patients with benign intracranial tumor.^3,6,8,13^ The same conclusion was drawn from the single population-based analysis on the matter.^14^ Treatment of these patients, however, is beyond the approval of the Food and Drug Administration. Likewise, evidence on the use of IV tissue plasminogen activator (t-PA) in patients with cavernoma is sparse, with 11 individual cases in two case reports^15,16^ and a single systematic analysis^17^ that yielded conflicting results. The aim of this study was to comprehend the risk of IV t-PA treatment in patients with intracranial tumor and cavernoma, in order to facilitate the clinician’s burden of taking the decision in this common but insufficiently respected constellation.

## Methods and patients

### Patient selection

Patients with evidence of intracranial tumor or cavernoma as well as the same number of age- and gender-matched control subjects, who received IV t-PA treatment at our comprehensive stroke center over a period of 7 years, were included in the analysis. Additionally, patients and control subjects were matched for the time of symptom-onset to the beginning of t-PA treatment to avoid concomitant bias on outcome and intracranial bleeding complications. The study was approved by the local institutional ethics committee (Medizinische Ethikkomission II der Medizinischen Fakultät Mannheim der Ruprecht-Karls-Universität Heidelberg), which did not demand informed consent for enrollment in the study.

### Clinical management and data acquisition

Thrombolytic therapy was administered using alteplase (Actilyse^®^) at a dosage of 0.9 mg/kg over a period of 60 min with 10% bolus injection. Diagnostic work up included MRI or conventional CT imaging before IV t-PA treatment, as well as MRI scans following treatment, including DWI, T1- and T2-weighted studies, fluid attenuation recovery (FLAIR) and T2*. Where an MRI was not performable for any particular reason, a CT was arranged. Hemorrhagic transformation was defined as parenchymal hemorrhage of the infarct territory itself, while symptomatic hemorrhage was defined as any deterioration of symptoms following intracranial hemorrhage for higher sensitivity. Besides clinical history, documented imaging reports were reviewed consistently for each participant and the original imaging data were assessed once more. Nine patients were excluded from analysis due to disagreement on tumor or cavernoma diagnosis.

### Statistical analysis

Statistical analysis was performed using SPSS Version 22. Differences in frequency of categorical variables were reviewed using the Chi-square test. Baseline and outcome values were compared using the t test or Mann–Whitney U Test for independent variables.

## Results

Thirty-four patients with intracranial tumor and cavernoma as well as the same number of control subjects were included in the analysis. Important baseline characteristics, as specified in Table 1, showed no differences when comparing both groups. The mean duration of stay was 10 days for the patient group and 9 days for the control group, which seems comfortably sufficient to identify intracranial hemorrhage due to alteplase therapy.

**Table 1.** Baseline characteristics of patients with intracranial tumor/cavernoma and control subjects. Some patients may have competing vascular risk factors and stroke etiologies, referring to the ASCO score, while stroke etiology could not be assessed sufficiently in six patients and two control subjects.

	Patients with intracranial tumor/cavernoma	Control subjects	*p* value
Age (years)	78 (±12)	78 (±12)	1.000
Female/male (*n*)	24/10	24/10	1.000
Duration of stay (days)	9.6 (±5.7)	8.8 (±4.3)	0.525
Time between symptom-onset to beginning of rt-PA treatment (min)	182 (±91)	181 (±94)	0.967
Stroke etiology (*n*)			
Atherosclerosis	5	8	0.548
Small-vessel disease	3	3	1.000
Cardiac disease	19	17	0.436
Other and unidentified etiology	7	12	0.412
Vascular risk factors (*n*)			
Diabetes	13	5	0.053
Hypertension	30	30	1.000
Smoking	5	3	0.709
Hyperlipidemia	21	13	0.089
Premedication (*n*)			
Antiplatelet therapy	17	10	0.136
Anticoagulant therapy	4	4	1.000
No preventive treatment	12	19	0.088

rt-PA, recombinant tissue plasminogen activator.

**Figure 1.**
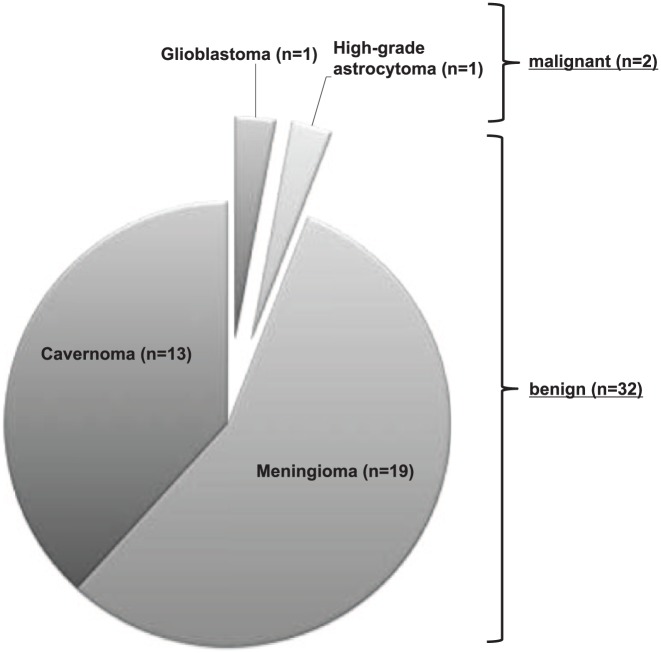
Specification of tumors and cavernomas considered either benign (94%) or malignant (6%).

A total of 32/34 (94%) of the masses observed were meningioma or cavernoma and considered benign. Only 2/34 (6%) of the patients presented with malignant intracranial neoplasm (see Figure 1). Both diagnoses were made after the MRI scan subsequent to acute stroke treatment and the presenting stroke mimic was the first manifestation of the underlying malignancy in these patients. Clinicians were therefore unaware of the tumor diagnosis prior to thrombolysis. Meningioma and cavernoma were documented in 22/34 (65%) patients before t-PA treatment was initiated and the therapy was started in the form of an expanded access after the patient’s informed approval and careful evaluation. None of these patients received special treatment of meningioma or cavernoma, respectively, prior to thrombolysis. In the other patients the intracranial tumor or cavernoma was not detected on initial CT imaging and were unknown. For more details on imaging, see Table 2.

**Table 2.** Imaging details of patients with intracranial tumor/cavernoma and control subjects.

	Patients with intracranial tumor/cavernoma	Control subjects	*p* value
MRI imaging before treatment [*n* (%)]	9/34 (26%)	1/34 (3%)	0.013
MRI imaging after treatment [*n* (%)]	29/34 (85%)	25/34 (74%)	0.369
Diagnosis of tumor/cavernoma before t-PA treatment [*n* (%)]	22/34 (65%)		
Secondary intracranial hemorrhage [*n* (%)]	9 (26%)	6 (18%)	0.560
Maximum transversal area of			
Infarct territory (cm^2^/± SD)	5.44 ± 10.38	7.42 ± 10.81	0.472
Meningioma (cm^2^/± SD)	1.70 ± 1.81		
Cavernoma (cm^2^/± SD)	0.28 ± 0.12		
Glioblastoma (cm^2^)	6		
High-grade astrocytoma (cm^2^)	2.25		
Range of transversal area of			
Cavernoma (cm^2^)	0.15–0.54		
Meningioma (cm^2^)	0.18–5.9		
Localization of cavernoma			
Infratentorial (*n*)	3		
Cortical (*n*)	6		
Subcortical (*n*)	5		
Multiple cavernoma (*n*)	1/13		
Symptomatic cavernoma (*n*)	1/13		
Multiple meningioma (*n*)	1/19		
Symptomatic meningioma (*n*)	3/19		

Except for one case, where hemorrhage occurred that was neither infarct nor tumor related in a patient with high-grade astrocytoma, mostly decent hemorrhagic transformation of the infarct territory was reason for secondary intracranial hemorrhage. The incidence observed in control subjects (9/34, 26%) did not differ from the incidence observed in patients (6/34, 18%; *p* = 0.56). Hemorrhage was considered symptomatic only in one patient, but in five control subjects (*p* = 0.20). Likewise, outcome parameters including death, as shown in Table 3, showed no difference between patients and control subjects. The prevalence of stroke mimics did not differ significantly between patients (8/34, 24%) and control subjects (5/34, 15%; *p* = 0.54). However, both patients with malignant intracranial neoplasm were stroke mimics due to tumor-related symptoms. Also, 3/6 (50%) stroke mimics observed in patients with meningioma or cavernoma can be attributed to focal seizures provoked by the corresponding intracranial mass.

**Table 3.** Outcome parameters of patients and matched control subjects. NIHSS, mRS and Barthel scale are given by 25%/75% percentile. Statistical analysis revealed no significant differences between the two groups.

	Patients with intracranial tumor/cavernoma	Control subjects	*p* value
Death (*n* [%])	2 (6%)	3 (9%)	1.000
NIHSS (National Institutes of Health Stroke Scale)			
Admission	7 (5/16)	10 (4/17)	0.782
Day 3	6 (2/12)	5 (1/15)	0.504
Dismissal	7 (1/15)	5 (0/17)	0.499
mRS (modified Rankin Scale)			
Admission	5 (4/5)	5 (3/5)	0.973
Day 3	4 (3/5)	4 (1/5)	0.460
Dismissal	4 (3/5)	4 (1/5)	0.468
Barthel scale			
Admission	35 (5/75)	20 (5/65)	0.628
Day 3	50 (15/75)	55 (5/90)	0.609
Dismissal	45 (5/80)	55 (5/100)	0.504

## Discussion

We did not observe any bleeding complications associated with the use of t-PA in patients with benign intracranial tumor or cavernoma. Our data are therefore in line with previously published observations of either intra-arterial or systemic thrombolysis for ischemic stroke^3–6,12–14^ or myocardial infarction^7,8^ in patients with benign intracranial tumor, and also contribute to the positive safety results reported in patients with cavernoma.^15,17^ However, we did observe independent intracranial hemorrhage following thrombolysis in one of the two patients with malignant intracranial neoplasm. This reflects the results of an increased bleeding risk in malignant brain tumor-associated stroke yielded by population-based analysis,^14^ as well as by previous case reports that reported secondary intracranial hemorrhage in two of seven patients with malignant intracranial neoplasm (including this report).^9–12^ Nevertheless, the strong heterogeneity of these neoplasms in terms of size, localization and histopathology makes it difficult to determine general principles in acute stroke therapy. Moreover, the low number of patients reported with malignant intracranial neoplasms does not allow drawing final conclusions. Also, the pathophysiological basis for the commonly presumed increased intracranial bleeding risk has not been discussed so far. However, it is justified to speculate that tumorangiogenesis and neovascularization in synergy with tumor necrosis and vessel infiltration will strongly enhance the bleeding risk in patients with malignant intracranial neoplasm.

Except for one control subject, death was not associated with intracranial hemorrhage, but was due to extensive comorbidity and infarct itself in this cohort of elderly patients. The high incidence of symptomatic secondary intracranial hemorrhage in the control group requires explanation, but may be explained by our low-threshold definition of ‘symptomatic’ for study purposes, as well as the advanced age and multi-morbidity of the patients.

Intracranial tumor may further promote the emergence of focal seizures or be accompanied by a focal neurological deficit itself and by doing so mimic an ischemic stroke. However, in our analysis the overall frequency of stroke mimics did not differ significantly between patients and control subjects. On the other hand, both patients with malignant intracranial neoplasm were stroke mimics. These results are in line with previously published reports and indicate a high incidence of stroke mimics in patients with malignant intracranial neoplasm, even if numbers are too low for statistical analysis.^6,9,10^ It should also be mentioned that a similar stroke mimic with symptomatic hemorrhage of a cavernoma after IV t-PA treatment was reported by Erdur and colleagues before, which highlights the necessity of further publication of safety results in these patients.^17^

To the best of our knowledge, this is the first case-controlled analysis so far evaluating the safety of off-label IV t-PA treatment for ischemic stroke in patients with concomitant evidence for intracranial tumor or cavernoma. However, the inherent limitations of our study approach, especially a potential selection bias and the heterogeneity of the patient group, must be considered. It also has to be noted that multimodal CT scan, including CT angiography and perfusion CT, was not always performed prior to thrombolysis. However, multimodal CT imaging including CT angiography and perfusion CT may be capable of identifying stroke mimics by the display of hyperperfusion and therefore further enhance the benefit–risk profile of thrombolysis in this cohort of patients. Furthermore, this study is limited to primary intracranial tumor and cavernoma and does not reflect the bleeding risk of secondary intracranial tumor following systemic thrombolysis. Also, secondary hemorrhage may conceal the underlying pathology on MRI after t-PA treatment and result in an underestimation of bleeding complications linked to an intracranial tumor or cavernoma.

Intra-arterial thrombectomy may be a feasible option, especially in patients with malignant intracranial neoplasm, in order to reduce the intracranial bleeding risk, but is still often not readily available and itself awaits further evaluation in this cohort of patients.

In conclusion, IV t-PA treatment in patients with meningioma and cavernoma appears to be relatively safe and reasonable, while the therapy should be avoided in patients with malignant intracranial neoplasm with blood–brain barrier disruption.
